# Identification of novel first-trimester serum biomarkers for early prediction of preeclampsia

**DOI:** 10.1186/s12967-023-04472-1

**Published:** 2023-09-18

**Authors:** Mingxi Liu, Yue Niu, Kongyang Ma, Peter C. K. Leung, Zi-Jiang Chen, Daimin Wei, Yan Li

**Affiliations:** 1 Center for Reproductive Medicine, Shandong University, Jinan, 250012 Shandong China; 2 Medical Integration and Practice Center, Shandong University, Jinan, 250012 Shandong China; 3 State Key Laboratory of Reproductive Medicine and Offspring Health, Shandong University, Jinan, 250012 Shandong China; 4 Centre for Infection and Immunity Studies, School of Medicine, The Sun Yat-sen University, Shenzhen, Guangdong China; 5 Department of Obstetrics and Gynecology, University of British Columbia, Vancouver, BC V5Z 4H4 Canada

**Keywords:** Prediction, Preeclampsia, First trimester, Serum, Biomarker

## Abstract

**Background:**

Preeclampsia (PE) is a leading cause of maternal and perinatal mortality and morbidity worldwide, but effective early prediction remains a challenge due to the lack of reliable biomarkers.

**Methods:**

Based on the extensive human biobank of our large-scale assisted reproductive cohort platform, the first-trimester serum levels of 48 cytokines, total immunoglobulins (Igs), anti-phosphatidylserine (aPS) antibodies, and several previously reported PE biomarkers [including placental growth factor (PlGF), soluble fms-like tyrosine kinase-1 (sFlt-1), and activin A] were measured in 34 women diagnosed with PE and 34 matched normotensive controls.

**Results:**

The PE group has significantly higher first-trimester serum levels of interleukin (IL)-2Rα, IL-9, tumor necrosis factor-β (TNF-β), RANTES, hepatocyte growth factor (HGF), total IgM, and total IgG, and aPS IgG optical density (OD) value, as well as lower first-trimester serum levels of PlGF and total IgA and aPS-IgG immune complexes (IC) OD value than the control group. Combining top five first-trimester serum biomarkers (total IgM, total IgG, PlGF, aPS IgG, and total IgA) achieved superior predictive value [area under the curve (AUC) and 95% confidence interval (CI) 0.983 (0.952–1.000), with a sensitivity of 100% and a specificity of 94.1%] for PE development compared to PlGF and PlGF/sFlt-1 independently [AUC and 95% CI 0.825 (0.726–0.924) and 0.670 (0.539–0.800), respectively].

**Conclusion:**

We identified novel first-trimester serum biomarkers and developed an effective first-trimester prediction model using immune-related factors and PlGF for PE, which could facilitate the development of early diagnostic strategies and provide immunological insight into the further mechanistic exploration of PE.

**Supplementary Information:**

The online version contains supplementary material available at 10.1186/s12967-023-04472-1.

## Introduction

Preeclampsia (PE) is a pregnancy-specific disease characterized by the de novo development of concurrent high blood pressure (≥ 140/90 mm Hg) and proteinuria (≥ 300 mg/L per 24 h) or other organ damage after 20 weeks of gestation. This condition affects 2–8% of pregnancies and is a major contributor to maternal and perinatal morbidity and mortality worldwide [1, 2]. However, clinical intervention for PE remains limited to passive, symptomatic treatment after symptom onset, with delivery as the sole definitive treatment. Notably, most maternal deaths are preventable and often result from missed or delayed diagnoses, underlining the need for early identification and intervention in PE cases [3]. Although low-dose aspirin utilization starting before 16 weeks of gestation could prevent the development of PE to some extent [4, 5], effective measures for early prediction of PE are still lacking; thus, numerous patients have missed golden opportunities for early intervention, which is largely due to the intricate admixture nature of this disease and a lack of effective first-trimester biomarkers.

Recent studies have supported the immune maladaptation hypothesis as the etiology of PE [6]. Dysregulated immune cells, such as regulatory T cells, macrophages, NK cells, and neutrophils, induce placental dysfunction, which is a “seed” of preeclampsia [7–9]. Previous studies have suggested that women conceiving through donor oocytes, intracytoplasmic single sperm injection, or with different partners are at higher risk of PE, implying an autoimmune mechanism underlying PE pathogenesis [9–11]. However, it remains unclear whether immune-related factors are dysregulated in the first-trimester serum of patients with preeclampsia and whether these factors hold promise as predictive biomarkers for this condition.

Our group previously demonstrated dysregulated cytokine profiles in women diagnosed with pregnancy-induced hypertension (PIH) as early as the first trimester, findings implying that several cytokines could be informative biomarkers for the early prediction of PIH [12]. Moreover, a meta-analysis of large cohort studies showed that among the primary clinical risk factors for PE, antiphospholipid antibody syndrome had the highest pooled PE rate [13]. Antiphospholipid antibodies have been reported to participate in multiple PE development processes, including placental microthrombogenesis [14], decidual acute atherosis [15], placental mitochondrial reactive oxygen species (ROS) production [16], and aberrant cell death with necrotic trophoblast debris release [17]. Antiphosphatidylserine/prothrombin antibodies (aPS/PT) are the most common type of antiphospholipid antibody in the serum of women developing PE [18, 19]. Furthermore, the presence of aPS/PT IgM has been suggested as a risk factor for endothelial dysfunction in women with PE [18]. However, the predictive value of aPS antibodies in PE development has received relatively little attention in current research.

The identification of PE-related maternal circulating factors, including placental growth factor (PlGF), soluble fms-like tyrosine kinase-1 (sFlt-1, also known as soluble VEGFR-1), vascular endothelial growth factor (VEGF), and activin A, has facilitated the prediction and diagnosis of PE in the second and third trimesters of pregnancy [20]. However, the imbalance of these factors in maternal circulation, including a high sFlt-1:PlGF ratio, has limited capacity to accurately predict PE at an earlier stage [21]. With advances in PE screening, a single relevant indicator (e.g., PlGF) is no longer considered sufficient for predicting PE effectively due to the heterogeneity and complexity of this disease [22]. It has been suggested that the addition of PlGF to multivariable models might be useful in increasing predictive performance [23]. Typically, the Fetal Medicine Foundation (FMF) competing-risks model incorporates several maternal clinical characteristics, mean arterial blood pressure, uterine artery pulsatility index on ultrasonography, and maternal circulating PlGF levels at 11–13 weeks of gestation, facilitating the prediction of PE [24]. However, more immunologic or inflammatory-related biomarkers need to be explored to improve overall screening performance for PE.

In this study, we hypothesized that there may be dysregulated maternal serum cytokine profiles and autoimmune antibodies in the first trimester of pregnancy of PE patients compared to normotensive controls. Based on the human biobank of our large-scale assisted reproductive cohort platform, serum samples collected at 11–13 weeks of gestation after in vitro fertilization (IVF) treatment, a high risk factor for PE [25], were subjected to profiling of 48 cytokines, autoimmune antibodies, and several previously reported PE biomarkers with the aim of identifying novel serum biomarkers and constructing an efficient predictive model for PE clinical management.

## Materials and methods

Detailed methods are provided in the online-only Additional file 1: Detailed Methods.

### Patients

**Fig. 1:**
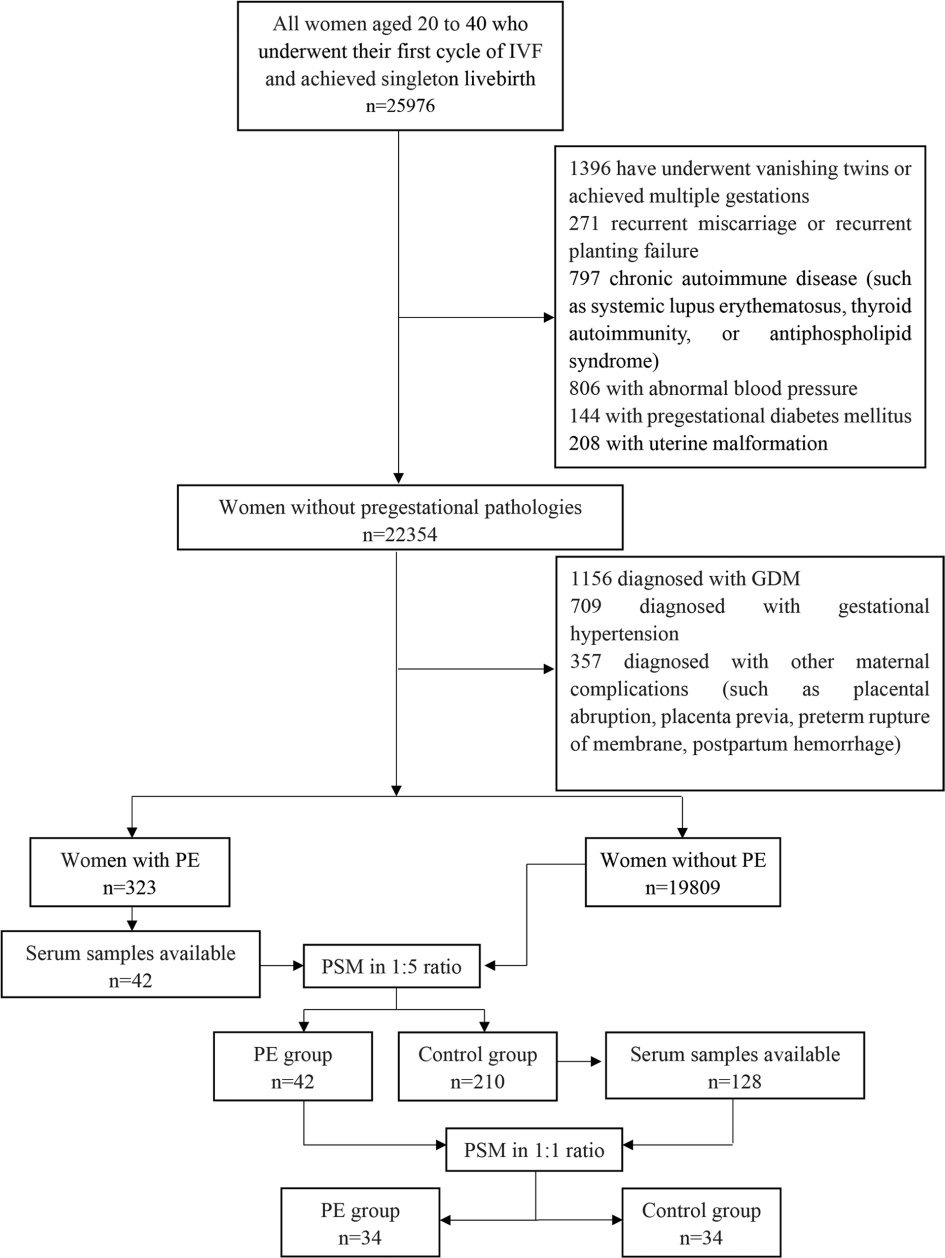
Flow diagram depicting participant screening and enrollment process. *IVF* in vitro fertilization, *APS* antiphospholipid syndrome, *BMI* body mass index, *GDM* gestational diabetes mellitus, *PE* preeclampsia, *PSM* propensity score matching

We included 34 women aged 20–40 years who underwent their first cycles of IVF with or without intracytoplasmic sperm injection (ICSI) and achieved singleton delivery between January 2015 and March 2020 in both the PE group and control group. Ethical approval for the use and analysis of blood samples and data from patients included in our study was obtained from the Institutional Ethical Committee of Medical Integration and Practice Center of Shandong University (Ethical Review No. SDULCLL2021-1-15). All participants provided informed written consent. Initially, a total of 25,976 women who achieved singleton pregnancy were screened for eligibility (Fig. 1), and none of them underwent vanishing twins or reduction of twins. Preimplantation genetic test cycles, donor oocyte cycles, or frozen-thawed oocyte cycles were excluded from this study. Patients were excluded from the study if they were diagnosed with uterine malformation, recurrent miscarriage (defined as three or more previous spontaneous pregnancy losses), recurrent implantation failure (failure to achieve a clinical pregnancy after three fresh or frozen cycles with good quality embryos), chronic autoimmune disease (such as systemic lupus erythematosus, thyroid autoimmunity, or antiphospholipid syndrome), preconceptional hypertension, preconceptional diabetes mellitus, or other diseases that may affect the inflammatory and immune processes. Additionally, women without available first-trimester serum samples were also excluded.

To reduce interference from other pregnancy complications, women who developed gestational diabetes mellitus (GDM), gestational hypertension, placental abruption, placenta previa, preterm rupture of membrane, and postpartum hemorrhage were also excluded. Blood samples of participants were collected at 11–13 gestational weeks after IVF treatment and subjected to serum preparation and storage in our extensive human biobank. The follow-up of pregnancy complications after IVF treatment were achieved basing on our large-scale assisted reproductive cohort platform.

The baseline characteristics of patients with available first-trimester serum samples were compared and matched using propensity score matching (PSM) approach to control for potential confounding bias. Maternal age, paternal age, body mass index, systolic pressure, diastolic pressure, preconceptional fasting glucose, gravidity, parity, diagnosis with polycystic ovary syndrome (PCOS), infertility cause, ovarian stimulation protocols, fertilization method, use of donor sperm, number of embryos transferred, embryo stage at transfer, endometrial thickness before embryo transfer, embryo transfer regimen, and embryo transfer time, which were weighted equally. The control group included 34 healthy women who were matched in a 1:1 ratio to the PE group based on the propensity score with a standard caliper width of 0.1. The study flow diagram is shown in Fig. 1.

PE was defined as a de novo development of concurrent high blood pressure (≥ 140/90 mm Hg) and proteinuria (≥ 300 mg/L per 24 h) or other maternal organ dysfunction, such as renal or liver involvement, neurological or hematological complications, or uteroplacental dysfunction (e.g. fetal growth restriction, abnormal umbilical artery Doppler waveform analysis, or stillbirth) [26]. PE was subclassified into early-onset PE (delivery at < 34 week’s gestation) and late-onset PE (delivery at ≥ 34 week’s gestation) [27].

### Sample collection and measurement of candidate biomarkers

First-trimester serum samples were collected from a total of 34 women diagnosed with PE and 34 matched normotensive controls. The levels of 48 cytokines, total immunoglobulins (IgA, IgM, and IgG), aPS autoantibodies (including aPS IgA, aPS IgM, and aPS IgG), aPS immune complexes (including aPS-IgA IC, aPS-IgM IC, and aPS-IgG IC), as well as previously identified PE biomarkers such as placental growth factor (PlGF), soluble fms-like tyrosine kinase-1 (sFlt-1), and activin A were measured in these samples.

The measurement of candidate biomarkers was performed using the Bio-Plex Pro Human Cytokine Screening Panel, 48-plex (Bio-Rad, #12007283) for cytokine analysis, and enzyme-linked immunosorbent assays (ELISA) for specific biomarker quantification. Detailed information regarding the sample collection procedure and the specific methods employed for measuring the candidate biomarkers can be found in the online-only Additional file 1: Detailed Methods.

### Statistical analysis

Statistical analysis was performed using SPSS 26.0 for Mac (IBM, Armonk, NY, USA) and GraphPad Prism 9.0 (GraphPad Software, San Diego, CA, USA). For continuous variables, normality was tested by the graphical use of histograms, Q–Q plots, and the Shapiro‒Wilk test; and data were presented as the means ± standard deviations (for normally distributed data) or medians with interquartile range (for nonnormally distributed data). Continuous variables with normal distributions were analyzed by student’s test, and those with nonnormal distributions were compared by the Mann–Whitney U test. For paired comparisons, the Wilcoxon paired test was used to compare significant differences. Categorical variables were presented as counts (percentages) and were compared using either the chi-square analysis or Fisher’s exact test. *P* < 0.05 were considered statistically significant, and cytokines with *P* < 0.05 were included in the Receiver Operating Characteristic (ROC) analysis. ROC curves were drawn to analyze the independent and combined values of specific cytokines and aPS antibodies in predicting PE.

## Results

### Baseline characteristics and pregnancy outcomes of the participants

In this study, 34 women who developed PE after IVF treatment and 34 matched normotensive controls after IVF treatment were included in the analysis. The baseline characteristics, including maternal age, paternal age, body mass index, blood pressure, preconceptional fasting glucose and hormonal parameters, pregnancy history, infertility causes, cycle characteristics of ovarian stimulation, and embryo culture and transfer, were comparable between the two groups (Additional file 1: Table S1).  The pregnancy outcomes are presented in Table 1. The risks of cesarean delivery (97.1% vs. 64.7%, *P* = 0.001) and preterm delivery (38.2% vs. 2.9%, *P* < 0.001) were higher in the PE group than in the control group. The PE group was associated with a lower birthweight [(3014.39 ± 962.27) g vs. (3419.12 ± 412.95) g, *P* = 0.031] and a higher rate of low birthweight of newborns (30.3% vs. 2.9%, *P* = 0.003) compared to the control group. There were no significant differences in the risks of macrosomia, small for gestational age (SGA), and large for gestational age (LGA) between these two groups.

**Table 1:** Pregnancy outcomes of the participants

Characteristics	Control (N = 34)	PE (N = 34)	*P* Value
Delivery mode-no. (%)			0.001
Vaginal delivery	12 (35.3%)	1 (2.9%)	
Cesarean delivery	22 (64.7%)	33 (97.1%)	
Preterm delivery-no. (%)	1 (2.9%)	13 (38.2%)	< 0.001
Birthweight (g) -Mean ± SD	3419.12 ± 412.95	3014.39 ± 962.27	0.031
Low birthweight-no. (%)	1 (2.9%)	10 (30.3%)	0.003
Macrosomia-no. (%)	2 (5.9%)	4 (12.1%)	0.427
SGA-no. (%)	2 (5.9%)	5 (17.2%)	0.233
LGA-no. (%)	8 (23.5%)	9 (31.0%)	0.504

* PE* preeclampsia, *SGA* small-for-gestation-age, *LGA* large-for-gestation-age

### Profiling of first-trimester serum cytokines identified dysregulation of five cytokines associated with PE development

**Fig. 2:**
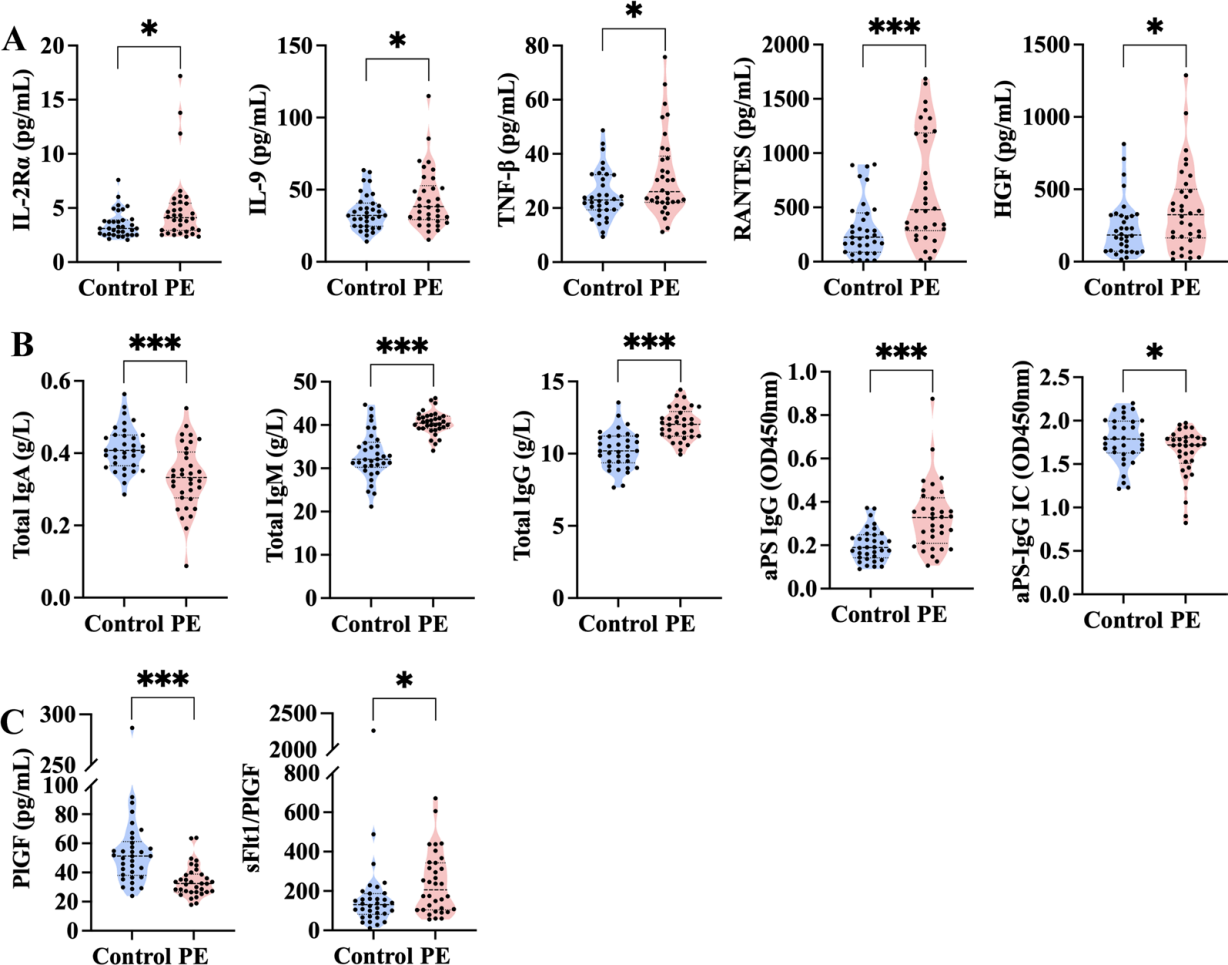
Violin plots illustrate significantly different first-trimester serum biomarkers between the PE group and the normotensive control group. **A** Violin plots of five first-trimester serum cytokines, including IL-2Rα, IL-9, TNF-β, RANTES, and HGF, for the PE group (n = 34) and normotensive control group (n = 34). **B** Serum levels of five first-trimester serum antibodies (total IgA, IgM, and IgG, aPS IgG, and aPS-IgG IC) for the PE group (n = 34) and normotensive control group (n = 34). **C** Serum concentrations of PlGF and sFlt-1/PlGF in the PE group (n = 34) and normotensive control group (n = 34). Data are presented as the mean ± standard deviation or median (interquartile range). *P < 0.05; ** *P* < 0.01; ***P < 0.001. *Ig* immunoglobulin, *IC* immune complex, *IL-2Rα* interleukin-2Rα, *IL-9* interleukin-9, *TNF-β* tumor necrosis factor-β, *HGF* hepatocyte growth factor, *aPS* anti-phosphatidylserine, *PlGF* placental growth factor, *sFlt-1* soluble fms-like tyrosine kinase 1

The serum levels of 46 factors in the PE and control groups are summarized in Table 2. Among the 46 cytokines, five cytokines, including IL-2Rα, IL-9, TNF-β, RANTES, and HGF, were higher in the PE group than in the control group. In addition, violin plots were generated for these five biomarkers (Fig. 2A).

**Table 2:** **Cytokine profile in first-trimester serum**

Cytokine (pg/mL)	Control (N = 34)	PE (N = 34)	*P* value	Cytokine (pg/mL)	Control (N = 34)	PE (N = 34)	*P* value
IL-1β	0.75 (0.59, 1.11)	0.86 (0.63, 1.50)	0.253	G-CSF	399.98 (126.85, 829.60)	255.96 (73.95, 645.37)	0.204
IL-1α	11.94 (10.44, 15.21)	12.55 (10.14, 15.83)	0.796	M-CSF	1.81 (1.52, 1.94)	1.81 (1.60, 2.16)	0.383
IL-1ra	182.92 (164.35, 208.81)	196.10 (154.56, 262.78)	0.689	GM-CSF	0.83 (0.65, 1.15)	0.83 (0.65, 1.17)	0.995
IL-2	1.77 (1.50, 1.91)	1.65 (1.53, 2.00)	0.692	LIF	17.93 (14.73, 23.11)	18.28 (15.45, 25.30)	0.745
*IL-2Rα	3.5 ± 1.23	4.91 ± 3.26	0.023	SCF	13.54 (7.59, 27.51)	13.69 (9.56, 20.33)	0.787
IL-3	0.72 (0.66, 0.85)	0.76 (0.66, 0.92)	0.692	Eotaxin	1.85 (1.32, 3.88)	1.94 (1.14, 4.31)	0.951
IL-4	1.45 (1.21, 1.67)	1.29 (1.13, 1.60)	0.217	MIP-1α	16.84 (4.05, 37.04)	8.53 (1.86, 40.02)	0.524
IL-5	8.37 (7.80, 12.77)	8.37 (6.10, 11.69)	0.513	MIP-1β	37.09 (25.71, 97.07)	43.37 (29.90, 96.30)	0.704
IL-6	0.68 (0.60, 1.23)	0.65 (0.60, 0.95)	0.468	FGF Basic	15.45 ± 4.07	17.16 ± 4.23	0.095
IL-7	6.63 (6.06, 7.74)	6.63 (6.06, 7.74)	0.488	MCP-1	2.05 (1.83, 2.37)	2.05 (1.83, 2.82)	0.726
IL-8	19.37 (2.26, 82.16)	8.76 (2.23, 49.89)	0.606	MCP-3	1.22 (0.99, 1.53)	1.27 (0.99, 1.59)	0.990
*IL-9	34.58 ± 12.42	43.42 ± 20.74	0.037	β-NGF	1.77 ± 0.29	1.77 ± 0.23	0.946
IL-10	3.09 (2.86, 3.32)	3.09 (2.86, 3.37)	0.588	*RANTES	303.17 ± 266.21	670.64 ± 511.61	0.001
IL-12(p40)	26.22 (22.45, 30.84)	23.90 (20.11, 33.14)	0.416	SDF-1α	60.77 (42.57, 107.66)	63.53 (46.01 ,112.12)	0.556
IL-12(p70)	2.14 (2.06, 2.14)	2.08 (2.01, 2.14)	0.117	PDGF-BB	50.17 (27.83, 236.28)	97.99 (39.05, 447.97)	0.262
IL-13	1.08 (0.92, 1.12)	1.00 (0.92, 1.24)	0.650	GRO-α	115.22 (105.73, 133.88)	117.51 (108.15, 134.90)	0.457
IL-16	9.01 (6.93, 12.53)	10.13 (7.31, 21.04)	0.394	*HGF	233.76 ± 192.31	360.28 ± 292.57	0.039
IL-17	3.51 (3.10, 4.29)	3.69 (3.10, 4.44)	0.681	IP-10	15.23 (10.33, 23.44)	15.62 (11.06, 28.91)	0.447
IL-18	2.00 (1.73, 2.56)	2.43 (1.82, 2.97)	0.152	CTACK	3.63 (3.28, 3.99)	3.55 (3.28, 3.99)	0.569
IFN-α2	5.12 (4.50, 5.30)	4.74 (4.16, 5.66)	0.543	MIF	22.74 (12.12, 38.11)	32.20 (15.86, 88.95)	0.080
IFN-γ	1.47 (1.28, 1.62)	1.56 (1.35, 1.74)	0.137	MIG	12.59 (8.69, 28.07)	15.91 (9.46, 31.45)	0.387
TNF-α	7.43 ± 1.71	7.12 ± 1.35	0.419	SCGF-β	3642.80 (1083.20, 9553.74)	2012.10 (1009.60, 7772.50)	0.528
* TNF-β	24.9 ± 9.15	31.79 ± 15.33	0.029	TRAIL	1.96 (1.56, 3.01)	2.13 (1.67, 2.94)	0.361

Data are presented as the mean ± standard deviation or median (interquartile range). P < 0.05 was considered statistically significant and indicated by an asterisk

*IL-1β* interleukin-1β, *IL-1α* interleukin-1α, *IL-1ra* interleukin-1ra, *IL-2* interleukin-2, *IL-2Rα* interleukin-2Rα, *IL-3* interleukin-3, *IL-4* interleukin-4, *IL-5* interleukin-5, *IL-6* interleukin-6, *IL-7* interleukin-7, *IL-8* interleukin-8, *IL-9* interleukin-9, *IL-10* interleukin-10, *IL-12 (p40)* interleukin-12(p40), *IL-12(p70)* interleukin-12(p70), *IL-13* interleukin-13, *IL-16* interleukin-16, *IL-17* interleukin-17, *IL-18* interleukin-18, *IFN-α2* interferon-α2, *IFN-γ* interferon-γ, *TNF-α* tumor necrosis factor-α, *TNF-β* tumor necrosis factor-β, *G-CSF* granulocyte colony-stimulating factor, *M-CSF* macrophage colony-stimulating factor, *GM-CSF* granulocyte-macrophage colony-stimulating factor, *LIF* leukemia inhibitory factor, *SCF* stem cell factor, *Eotaxin* MIP-1α,macrophage inflammatory protein-1α, *MIP-1β* macrophage inflammatory protein-1β, *FGF basic* basic fibroblast growth factor, *MCAF(MCP-1)* monocyte chemoattractant activating factor or monocyte chemotactic protein-1, *MCP-3* monocyte chemotactic protein-3, *β-NGF* nerve growth factor-β, *SDF-1α* stromal cell derived factor-1α, *PDGFBB* platelet-derived growth factor-BB, *GRO-α* growth related oncogene-α, *HGF* hepatocyte growth factor, *IP-10* interferon inducible protein-10, *CTACK* cutaneous T-cell attracting chemokine, *MIF* mifepristone, *MIG* gamma-interferon-induced monokine, *SCGF-β* stem cell growth factor-β, *TRAIL* TNF-related apoptosis-inducing ligand

**Fig. 3:**
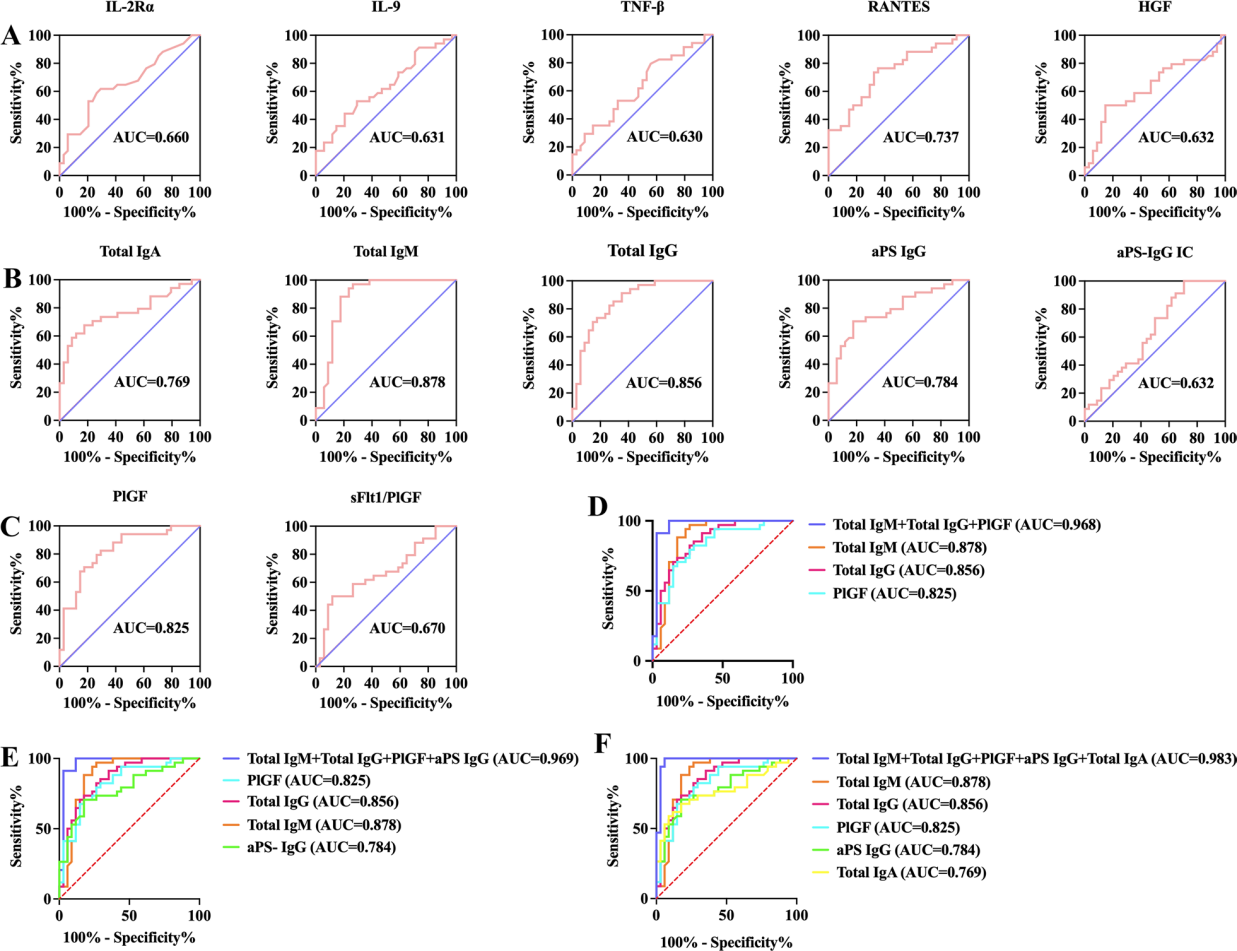
ROC curve evaluations of first-trimester serum biomarkers for PE. **A** ROC curves for five first-trimester serum cytokines: IL-2Rα (AUC: 0.660, 95% CI 0.529–0.790, *P* = 0.023), IL-9 (AUC: 0.631, 95% CI 0.499–0.763, *P* = 0.037), TNF-β (AUC: 0.630, 95% CI 0.497–0.762, *P* = 0.028), RANTES (AUC: 0.737, 95% CI 0.619–0.855, *P* = 0.001), and HGF (AUC: 0.632, 95% CI 0.497–0.767, *P* = 0.039). **B** ROC curves for five first-trimester serum antibodies: total IgA (AUC: 0.769, 95% CI 0.654–0.885, *P* < 0.001), total IgM (AUC: 0.878, 95% CI 0.786–0.969, *P* < 0.001), total IgG (AUC: 0.856, 95% CI 0.767–0.946, *P* < 0.001), aPS IgG (AUC: 0.784, 95% CI 0.675–0.893, P < 0.001), and aPS-IgG IC (AUC: 0.632, 95% CI 0.498–0.765, *P* = 0.035). **C** ROC curves for PlGF (AUC: 0.825, 95% CI 0.726–0.924, *P* < 0.001) and sFlt-1/PlGF (AUC: 0.670, 95% CI 0.539-0.800, *P* = 0.011). **D** The ROC curves for the combined analysis of the top three first-trimester serum biomarkers (including total IgM, total IgG, and PlGF). **E** ROC curves for the combined analysis of the top four first-trimester serum biomarkers total IgM, total IgG, PlGF, and aPS IgG). **F** ROC curves for the combined analysis of the top five first-trimester serum biomarkers (total IgM, total IgG, PlGF, aPS IgG, and total IgA) achieved an exceptional predictive value [AUC and 95% CI 0.983 (0.952-1.000), with a sensitivity of 100% and a specificity of 94.1%] for the development of PE. *ROC* receiver operating characteristic, *AUC* area under the curve, *CI* confidence interval, *IL-2Rα* interleukin-2Rα, *IL-9* interleukin-9, *TNF-β* tumor necrosis factor-β, *HGF* hepatocyte growth factor, *aPS* anti-phosphatidylserine, *Ig* immunoglobulin, *IC* immune complex, *PlGF* placental growth factor, *sFlt-1* soluble fms-like tyrosine kinase

The ROC curves for the five differentially expressed cytokines between the control and PE groups are shown in Fig. 3A. Serum levels of IL-2Rα (AUC: 0.660, 95% CI 0.529–0.790, *P* = 0.023), IL-9 (AUC: 0.631, 95% CI 0.499–0.763, *P* = 0.037), TNF-β (AUC: 0.630, 95% CI 0.497–0.762, *P* = 0.028), RANTES (AUC: 0.737, 95% CI 0.619–0.855, *P* = 0.001), and HGF (AUC: 0.632, 95% CI 0.497–0.767, *P* = 0.039) showed general predictive values for PE.

### Screening of first-trimester serum antibodies identified novel first-trimester serum biomarkers for PE

To evaluate the association between first-trimester serum antibodies and the development of PE, serum concentrations of total IgA, IgM, and IgG, as well as the OD values at 450 nm of aPS antibodies and aPS antibody ICs, were measured, as shown in Table 3. The serum concentrations of total IgM [(40.50 ± 2.65) g/L vs. (32.98 ± 5.47) g/L, *P* < 0.001] and total IgG [(12.06 ± 1.10) g/L vs. (10.27 ± 1.30) g/L, *P* < 0.001] and the OD values at 450 nm of aPS IgG [0.33 (0.21, 0.42) vs. 0.19 (0.14, 0.25), *P* < 0.001] were higher in the PE group than in the control group, while the PE group was also associated with a lower serum level of total IgA [(0.33 ± 0.09) g/L vs. (0.41 ± 0.06) g/L, *P* < 0.001] and the OD values at 450 nm of aPS-IgG IC [1.63 ± 0.28 vs. 1.77 ± 0.27, *P* = 0.035]. We also generated violin plots and ROC curves for serum levels of total IgA, IgM, and IgG, as well as aPS IgG and aPS-IgG IC, which are presented in Figs. 2B and 3B, respectively. ROC curve analysis showed favorable predictive values for total IgA (AUC: 0.769, 95% CI 0.654–0.885, *P* < 0.001), total IgM (AUC: 0.878, 95% CI 0.786–0.969, *P* < 0.001), total IgG (AUC: 0.856, 95% CI 0.767–0.946, *P* < 0.001), aPS IgG (AUC: 0.784, 95% CI 0.675–0.893, *P* < 0.001), and aPS-IgG IC (AUC: 0.632, 95% CI 0.498–0.765, *P* = 0.035). Of these, total IgM and total IgG had higher predictive values than PlGF (AUC: 0.825, 95% CI 0.726–0.924, *P* < 0.001) (Fig. 3C**)**, which provides new insights into the early prediction of women at high risk of PE.

**Table 3:** Antibody profile in first-trimester serum

Antibody (g/L)	Control (N = 34)	PE (N = 34)	*P* value
Total Igs (g/L)			
*Total IgA	0.41 ± 0.06	0.33 ± 0.09	< 0.001
*Total IgM	32.98 ± 5.47	40.50 ± 2.65	< 0.001
*Total IgG	10.27 ± 1.30	12.06 ± 1.10	< 0.001
aPS Antibodies (OD values at 450 nm)			
aPS IgA	0.72 ± 0.17	0.71 ± 0.15	0.879
aPS IgM	0.70 ± 0.21	0.71 ± 0.19	0.969
*aPS IgG	0.19 (0.14, 0.25)	0.33 (0.21, 0.42)	< 0.001
aPS-IgA IC	0.60 ± 0.18	0.57 ± 0.15	0.423
aPS-IgM IC	0.59 ± 0.21	0.60 ± 0.14	0.842
*aPS-IgG IC	1.63 ± 0.28	1.77 ± 0.27	0.035

Data are presented as the mean ± standard deviation and median (interquartile range). *P* < 0.05 was considered statistically significant and indicated by an asterisk

*Ig* immunoglobulin, *aPS* anti-phosphatidylserine, *OD* optical density, *IC* immune complex

### The first-trimester serum levels of PlGF and sFlt-1 are closely associated with PE development

To explore whether the levels of previously reported PE serum biomarkers in the first-trimester serum differ between the PE and control groups in ART pregnancies, first-trimester serum levels of PlGF, sFlt-1, and Activin A were assayed by ELISA for our samples with subsequent calculation of the sFlt-1/PlGF ratio and evaluation of their association with the development of PE. In the first-trimester of pregnancy, serum levels of PlGF were significantly lower in the PE group than in the control group [32.41 (26.60, 38.95) pg/mL vs. 51.25 (37.97, 61.19), *P* < 0.001], while sFlt-1/PlGF was higher in the PE group than in the control group [204.94 (103.34, 342.52) vs. 131.17 (81.74, 187.11), *P* = 0.016] (Fig. 2C and Additional file 1:  Table S2)

The ROC curve analysis revealed moderate predictive values for PlGF (AUC: 0.825, 95% CI 0.726–0.924, *P* < 0.001) and low predictive values for sFlt-1/PlGF (AUC: 0.670, 95% CI 0.539–0.800, *P* = 0.011) (Fig. 3C).

### Development of first-trimester serum predictive models for PE using immune-related factors and PlGF

Based on the predictive values of the above cytokines, autoimmune antibodies, and previously reported PE biomarkers, we selected the top five serum biomarkers (including total IgM, total IgG, PlGF, aPS IgG, and total IgA) according to the AUC area under the ROC curve and established combined predictive models, and the combinations of the first three, first four, and all five biomarkers yielded superior predictive values [AUC and 95% CI 0.968 (0.920–1.000), 0.969 (0.922–1.000), and 0.983 (0.952–1.000), respectively; sensitivities and specificities: 91.2% and 97.1%, 91.2% and 97.1%, 100% and 94.1%, respectively] for PE development, surpassing the predictive value of any single biomarker (Fig. 3D–F and Additional file 1:  Table S3). These findings suggest that the combined analysis of serum autoimmune antibodies and PlGF can improve the predictive value for PE development compared to PlGF alone.

Additionally, to explore whether the inclusion of early-onset PE cases influence the credibility of our model, we have examined the predictive power of our model for predicting early-onset PE (N = 6) and late-onset PE (N = 28), the AUCs and 95% CIs were 1.000 (1.000–1.000) and 1.000 (1.000–1.000), respectively, as illustrated in Additional file 1:  Fig. S1A and B) These data indicated that our model can robustly predict both early- and late-onset PE. However, caution should be exercised in regard to this conclusion considered its nature as stratified analysis and the small sample size.

## Discussion

Predicting preeclampsia in clinical practice remains challenging due to the lack of reliable first-trimester biomarkers. Although large biobanks and cohort platforms have facilitated research efforts, the identification of novel efficient biomarkers and evaluation of their clinical utility for early PE prediction are still warranted.

In this study, we observed significant differences in the serum levels of specific cytokines and autoimmune antibodies in women who developed PE and their normotensive controls during 11–13 weeks of gestation. The combined analysis of five significantly altered cytokines and antibodies, including PlGF, total IgA, total IgM, total IgG, and aPS IgG, achieved pronounced predictive values for PE. These findings indicate dysregulated immune-related cytokine and autoimmune antibody profiles in the first trimester serum of PE patients and suggest that the combined analysis of classical biomarkers such as PlGF and autoimmune antibodies can enhance the accuracy of early PE prediction.

PlGF, in addition to its involvement in the regulation of angiogenic/anti-angiogenic factors and inflammatory/anti-inflammatory mediators, also plays an immunomodulatory role during pregnancy [28, 29]. sFlt-1, primarily produced by syncytiotrophoblasts, is a biomarker of syncytiotrophoblast stress. [30] Multiple clinical studies have demonstrated that the sFlt-1/PlGF ratio facilitates the prediction and diagnosis of PE [31]. However, the effectiveness of PlGF in predicting PE in women undergoing IVF treatment remains unclear and warrants further investigation. Previous studies demonstrated that the sFlt-1/PlGF ratio achieved a higher predictive value for PE in mid-to-late pregnancy than either PlGF or sFlt-1 alone [31]. However, our study showed a higher predictive value for PlGF alone compared to the sFlt-1/PlGF ratio in early pregnancy. This may be due to syncytiotrophoblast stress and subsequent maternal endothelial dysfunction occurring after 13 weeks of gestation in middle or late pregnancy.

It is well established that the development of PE is closely associated with defective trophoblast invasion in early pregnancy [32]. Cytokines are key regulators of trophoblast invasion and participate in the inflammatory and immune regulation of PE development [33–35]. However, the literature presents conflicting evidence regarding the use of serum cytokines as early predictors of PE [36]. While some studies suggest potential utility of individual markers such as IL-8 [37], there is a clear need to explore a broader range of cytokines for a more robust prediction of PE. Although RANTES [38] and HGF [39] have been reported to promote human trophoblast cell invasion, their predictive values for PE development have rarely been explored. Furthermore, the contribution of cytokines such as IL-2Rα, IL-9, and TNF-β, which are known to be involved in inflammatory and autoimmune diseases [40], to the pathogenesis of PE remain unknown. In our study, we found that these five cytokines (RANTES, IL-2Rα, HGF, IL-9, and TNF-β) were significantly altered as early as the first trimester in women who later developed PE, and each of them demonstrated moderate predictive value. Further studies are needed to verify our findings and to identify additional candidate cytokines for early PE prediction.

Immune tolerance at the maternal-fetal interface is essential for establishing and maintaining a successful pregnancy [41]. It involves the interaction of placental cells with the maternal immune system, facilitating maternal-fetal immune dialogue [42]. It has been suggested that immune dysfunction can be identified before the clinical symptoms of PE manifest [37]; therefore, early detection of serum immunological markers for PE is of substantial clinical importance. Accumulating evidence suggests that IgA, IgM, and IgG play crucial roles in extensive immune responses. Serum levels of IgA, IgM, and IgG increase during the first trimester of pregnancy, highlighting the importance of Igs in the adaptive regulation of pregnancy [43].

Since immune dysregulation significantly contributes to hypertension disorders in pregnancy [44], many studies have investigated the relationship between IgA, IgM, and IgG expression and PE development. Research by Kestlerová et al. demonstrated higher serum levels of IgA, IgM, and IgG in women diagnosed with PE at delivery [45]. Increased IgM levels were observed in the kidneys of women diagnosed with PE [46]. Our study revealed reduced serum levels of total IgA as well as elevated serum levels of total IgM and IgG in the PE group compared to the normotensive control group at 11–13 weeks of gestation, which may be attributable to altered immune antibody expression. Previous studies have reported altered expression of autoantibodies, including angiotensin II type 1 receptor agonistic antibodies [47], anti-phospholipid antibodies [48], anticardiolipin antibodies [45], and aPS antibodies [18, 19], in the third trimester in women who developed PE. In our study, women in the PE group exhibited elevated first-trimester aPS IgG serum levels, while no significant differences in aPS IgA and IgM serum levels were observed. Whether other autoantibodies play roles in the development of PE need to be further studied.

B cells are also involved in immunoregulation in PE. Upon activation, B cells produce antibodies and cytokines that interact with T cells to modulate immune responses [14, 49, 50]. Regulatory B cells may play crucial roles in PE pathogenesis by maintaining the balance of T-helper (Th)1/Th2 and Th17/regulatory T cells [51]. Several studies have examined B lymphocytes in the context of PE. Matthiesen et al. found increased B lymphocyte serum levels in women who developed PE compared to normal pregnancies [52]. Liao et al. reported elevated peripheral blood memory B lymphocytes and plasma cell precursors in PE [53]. Our study suggests that alterations in serum IgA, IgM, and IgG levels may be due to quantitative or functional changes in B lymphocytes. The role of B lymphocytes in PE requires further investigation.

Despite extensive research efforts, the early prediction of PE remains elusive. A recent study showed that circulating cell-free RNA can predict PE between 5 and 16 weeks of gestation by establishing a logistic regression model that achieved an AUC of 0.99, with a sensitivity of 100% and a specificity of 85% [54]. Additionally, the study highlighted the significant contribution of the immune system to the observed changes in cell-free RNA in PE [54]. However, the technology involved in sample preparation, as well as the measurement and analysis of cell-free RNA, poses significant challenges [55]. Our study indicates that first-trimester autoimmune antibodies are altered in women diagnosed with PE, and the combined analysis of PlGF and autoimmune antibodies offers improved predictive values for PE. These findings underscore the potential role of autoimmune antibodies in the pathogenesis of PE, providing a cost-effective and noninvasive approach to assess the risk of PE. This could contribute to the early identification and management of women at high risk of developing PE. However, there were still some limitations in our study. Firstly, the small sample size, the retrospective nature of the study, and the lack of clinical validation necessitate future prospective studies with larger sample sizes to confirm our findings. Secondly, only first-trimester serum levels of cytokines and autoantibodies were assessed; further studies are warranted to examine dynamic changes in serum cytokines and autoantibodies throughout pregnancy. Lastly, accurate estimation of PE onset time is vital for clinical classifications and decisions, ensuring timely monitoring and intervention. However, our study did not investigate the onset time of PE, highlighting the need for future researches in this area.

### Conclusions

In conclusion, our findings reveal that in the first trimester of women who later developed PE, serum levels of cytokines, including HGF, IL-2Rα, IL-9, RANTES, and TNF-β, as well as autoimmune antibodies, such as total IgM, total IgG, and aPS IgG, were increased, while the level of total IgA antibody decreased. Furthermore, we observed lower serum levels of PlGF and a higher ratio of serum sFlt-1/PlGF in the PE group. Combined models incorporating serum PlGF and autoimmune antibodies (including total IgA, total IgM, total IgG, and aPS IgG) achieved high predictive values for PE.

### Supplementary Information

**Additional file 1: Detailed Methods. Table S1.** Baseline characteristics of the participants. **Table S2.** Classical serum biomarker levels in first-trimester serum of PE and normotensive controls. **Table S3.** ROC analysis of the predictive/diagnostic value of early pregnancy biomarkers for PE. **Figure S1.** The ROC curve for early- and late-onset PE.

## Data Availability

The datasets used and/or analysed during the current study are available from the corresponding author on reasonable request.

## Abbreviations

aPS: Anti-phosphatidylserine
AUC: Area under the curve
CI: Confidence interval
ELISA: Enzyme-linked immunosorbent assay
FMF: Fetal Medicine Foundation
HGF: Hepatocyte growth factor
LGA: Large for gestational age
IL-2Rα: Interleukin-2Rα
IL-9: Interleukin-9
IVF: In vitro fertilization
ICSI: Intracytoplasmic sperm injection
IC: Immune complex
Ig: Immunoglobulin
GDM: Gestational diabetes mellitus
OD: Optical density
PE: Preeclampsia
PIH: Pregnancy-induced hypertension
PCOS: Polycystic ovary syndrome
PlGF: Placental growth factor
PSM: Propensity score matching
PT: Prothrombin antibodies
ROS: Reactive oxygen species
ROC: Receiver operating characteristic
SGA: Small for gestational age
sFlt-1: Soluble fms-like tyrosine kinase-1
Th: T-helper
TNF-β: Tumor necrosis factor-β
VEGF: Vascular endothelial growth factor
