# Supplementary material for: Identification of novel first-trimester serum biomarkers for early prediction of preeclampsia
Source: J Transl Med. 2023 Sep 18;21:634. doi: 10.1186/s12967-023-04472-1 (PMC10506221; doi:10.1186/s12967-023-04472-1)
Supplement: Supplementary file 1 — Additional file 1: Detailed Methods. Table S1. Baseline characteristics of the participants. Table S2. Classical serum biomarker levels in first-trimester serum of PE and normotensive controls. Table S3. ROC analysis of the predictive/diagnostic value of early pregnancy biomarkers for PE. Figure S1. The ROC curve for early- and late-onset PE. [file 12967_2023_4472_MOESM1_ESM.docx]

**Additional file**

**For**

**Identification of Novel First-Trimester Serum Biomarkers for Early Prediction of Preeclampsia**

Mingxi Liu^1,2,3†^, Yue Niu^1,2,3†^, Kongyang Ma^4†^, Peter C.K. Leung^5^, Zi-Jiang Chen^1,3^, Daimin Wei^1,2,3*^, Yan Li^1,2,6*^

**Lists of Additional file**

[Detailed Methods 3](#_Toc11887)

[Additional file Tables 6](#_Toc27655)

[Additional file Figure 11](#_Toc28946)

# Detailed Methods

**Sample Collection**

Blood samples were collected at 11–13 gestational weeks after IVF treatment. Approximately 6 ml of fasting blood samples were collected into evacuated tubes by venipuncture from each participant. Serum was prepared from whole blood after blood coagulation and centrifugation. All serum samples were stored at -80 °C until measurement.

Baseline characteristics and IVF cycle information were obtained from IVF clinical records. Data on obstetrical and perinatal complications were collected by timely telephone follow-up by trained nurses. The diagnosis was generally based on the obstetric discharge records and neonate birth documents.

**Cytokine Profiling**

Cytokine profiling was measured using the Bio-Plex Pro Human Cytokine Screening Panel, 48-plex (Bio-Rad, #12007283), which allowed for the analysis of 48 cytokine and chemokine cell signaling molecules. These including Interleukin (IL)-1β, IL-1α, IL-1ra, IL-2, IL-2Rα, IL-3, IL-4, IL-5, IL-6, IL-7, IL-8, IL-9, IL-10, IL-12 (p40), IL-12 (p70), IL-13, IL-15, IL-16, IL-17A, IL-18, interferon-α2 (IFN-α2), interferon-γ (IFN-γ), tumor necrosis factor-α (TNF-α), tumor necrosis factor-β (TNF-β), granulocyte colony-stimulating factor (G-CSF), macrophage colony-stimulating factor (M-CSF), granulocyte-macrophage colony-stimulating factor (GM-CSF), leukemia inhibitory factor (LIF), stem cell factor (SCF), vascular endothelial growth factor (VEGF), Eotaxin, macrophage inflammatory protein-1α (MIP-1α), macrophage inflammatory protein-1β (MIP-1β), basic fibroblast growth factor (FGF basic), monocyte chemotactic protein-1 (MCP-1) or monocyte chemoattractant activating factor (MCAF), monocyte chemotactic protein-3 (MCP-3), nerve growth factor-β (β-NGF), RANTES, stromal cell derived factor-1α (SDF-1α), platelet-derived growth factor-BB (PDGFBB), growth related oncogene-α (GRO-α), hepatocyte growth factor (HGF), interferon inducible protein-10 (IP-10), cutaneous T-cell attracting chemokine (CTACK), mifepristone (MIF), gamma-interferon-induced monokine (MIG), stem cell growth factor-β (SCGF-β), and TNF-related apoptosis-inducing ligand (TRAIL).This high-performance, broad screening panel consists of a biologically relevant collection of adaptive immunity cytokines, pro-inflammatory cytokines, and anti-inflammatory cytokines all in a single well. In our study, the levels of IL-15 and VEGF are not available for comparison, since these two factors were undetectable in most serum samples.

**Enzyme-linked immunosorbent assay (ELISA)**

The serum levels of aPS autoantibodies [aPS immunoglobulin (Ig) G, aPS IgM, and aPS IgA] as well as standard curve were measured by ninety-six-well high-affinity binding microplates (514201, NEST) which coated with 60 μL of bovine serum albumin (BSA)-phosphatidylserine conjugate (CPB881Ge11, Cloud-Clone Corp) or purified anti-human IgG Fc antibody (410701, Biolegend) overnight at 4 °C. After blocking with 100 μl of 0.5% (w/v) gelatin (G8061, Solarbio) and 0.5% (w/v) BSA (A8020, Solarbio) for 1 h at room temperature and being washed three times, duplicated serum samples at a final dilution of 1:200 were added (purified anti-human igG Fc 0.5 μg/μL and dilute at half gradient to 0.488 ng/μL were set to manufacture standard curve) for 2 h at room temperature. The plates were then washed and incubated with the detection antibodies, respectively (HRP goat anti-human IgA antibody, 411002, BioLegend; biotin anti-human IgM antibody, 314504, BioLegend; biotin anti-human IgG Fc antibody, 410718, BioLegend). The standard antibody for total IgG was obtained from human IgG (SP001, Solarbio) diluted in PBS with different gradients.

For the detection of aPS immune complexes (ICs) [aPS-IgG IC, aPS-IgM IC, and aPS-IgA IC], anti-PS antibody (PAB881Ge01, Cloud-clone Corp. China) were coatedand serum samples were added. HRP goat anti-human IgA antibody/biotin anti-human IgG plus HRP-streptavidin/biotin anti-human IgM plus HRP-streptavidin/biotin anti-mouse IgG were used to detect aPS-IgA IC, aPS-IgM IC, and aPS-IgG IC, respectively.

Negative control (prepared with coating buffer without antigen) and positive control (systemic Lupus Erythematosus sera) were applied for excluding the nonspecific binding of and false positive results. The optical density (OD) values were read at 450 nm by a BioTek Epoch multiwavelength spectrum microplate. The regression equation derived from the standard curve was Y=0.0151x-0.0035, R^2^=0.9956. The levels of serum biomarkers of preeclampsia in ELISA were determined using the standard curve.

Commercial ELISA kits (Human PlGF Quantikine ELISA Kit, DPG00, R&D; Human VEGFR1/Flt-1 Quantikine ELISA Kit, DVR100C, R&D; Human/Mouse/Rat Activin A Quantikine ELISA Kit, DAC00B, R&D) were used to assay serum levels of previously reported biomarkers of PE: Activin A, PlGF, and sFlt-1.

# Additional file Tables

**Table S1. Baseline characteristics of the participants.**

| Characteristics | Control (N = 34) | PE (N = 34) | *P* value | |
| --- | --- | --- | --- | --- |
| Maternal age (yr)-Mean ± SD | 30.00 ± 3.97 | 30.06 ± 4.57 | 0.955 |  |
| Paternal age (yr)-Mean ± SD | 31.29 ± 4.62 | 31.32 ± 5.61 | 0.981 |  |
| Body mass index (kg/m²) -Mean ± SD | 24.31 ± 3.35 | 23.89 ± 3.65 | 0.625 |  |
| Blood pressure (mmHg)-Mean ± SD |  |  |  |  |
| Systolic pressure | 114.65 ± 11.86 | 113.74 ± 12.15 | 0.755 |  |
| Diastolic pressure | 71.06 ± 8.51 | 71.21 ± 8.48 | 0.943 |  |
| Preconceptional fasting glucose (mmol/L) -Mean ± SD | 5.12 ± 0.43 | 5.15 ± 0.46 | 0.785 |  |
| Baseline hormonal parameters |  |  |  |  |
| FSH (IU/L) -Mean ± SD | 6.09 ± 1.66 | 6.21 ± 1.39 | 0.744 |  |
| LH (IU/L) -Mean ± SD | 6.27 ± 4.72 | 6.37 ± 5.21 | 0.933 |  |
| E2 (pg/mL) -Mean ± SD | 33.45 ± 19.65 | 39.83 ± 27.20 | 0.272 |  |
| Total T (ng/dL) -Mean ± SD | 26.10 ± 11.19 | 28.49 ± 16.54 | 0.487 |  |
| Preconceptional TSH (μIU/mL) -Mean ± SD | 2.39 ± 0.90 | 2.40 ± 1.04 | 0.989 |  |
| Gravidity ≥ 1-no. (%) | 17 (50.0%) | 17 (50.0%) | >0.999 |  |
| Parity ≥ 1-no. (%) | 6 (17.6%) | 5 (14.7%) | 0.742 |  |
| Diagnosed with PCOS-no. (%) | 12 (35.3%) | 8 (23.5%) | 0.287 |  |
| Infertility causes-no. (%) |  |  | 0.533 |  |
| Pelvic factor | 10 (29.4%) | 14 (41.2%) |  |  |
| Male factor | 1 (2.9%) | 1 (2.9%) |  |  |
| Other factors | 1 (2.9%) | 3 (8.8%) |  |  |
| Mixed factors | 22 (64.7%) | 16 (47.1%) |  |  |
| Ovarian stimulation protocols-no. (%) |  |  | 0.700 |  |
| Long agonist protocol | 25 (73.5%) | 21 (61.8%) |  |  |
| Short agonist protocol | 4 (11.8%) | 5 (14.7%) |  |  |
| GnRH antagonist protocol | 5 (14.7%) | 7 (20.6%) |  |  |
| Other protocols | 0 | 1 (2.9%) |  |  |
| Fertilization method -no. (%) |  |  | 0.141 |  |
| IVF | 16 (47.1%) | 23 (67.6%) |  |  |
| ICSI | 17 (50.0%) | 11 (32.4%) |  |  |
| Half IVF/half ICSI | 1 (2.9%) | 0 |  |  |
| Use of donor sperm -no. (%) | 0 | 2 (5.9%) | 0.493 |  |
| Number of embryos transferred -no. (%) |  |  | 0.171 |  |
| 1 | 31 (91.2%) | 27 (79.4%) |  |  |
| 2 | 3 (8.8%) | 7 (20.6%) |  |  |
| Embryo stage at transfer -no. (%) |  |  | 0.709 |  |
| Cleavage-stage embryo | 3 (8.8%) | 5 (14.7%) |  |  |
| Blastocyst embryo | 31 (91.2%) | 29 (85.3%) |  |  |
| Endometrial thickness before embryo transfer (mm) -Mean ± SD | 9.65 ± 1.76 | 10.03 ± 2.11 | 0.420 |  |
| Embryo transfer regimen -no. (%) |  |  | 0.525 |  |
| Fresh embryo transfer | 5 (14.7%) | 7 (20.6%) |  |  |
| Frozen embryo transfer | 29 (85.3%) | 27 (79.4%) |  |  |
| Embryo transfer time -no. (%) |  |  | 0.080 |  |
| 2015-2016 | 5 (14.7%) | 11 (32.4%) |  |  |
| 2017-2018 | 25 (73.5%) | 16 (47.1%) |  |  |
| 2019-2020 | 4 (111.8%) | 7 (20.6%) |  |  |

Abbreviations: PE, preeclampsia; FSH, follicle-stimulating hormone; LH, luteinizing hormone; E2, estradiol; T, testosterone; TSH, thyroid-stimulating hormone; AFC, antral follicle count; PCOS, polycystic ovary syndrome; IVF, in vitro fertilization; ICSI, intracytoplasmic sperm injection.

**Table S2. Classical serum biomarker levels in first-trimester serum of PE and normotensive controls.**

| Cytokine | Control (N = 34) | PE (N = 34) | *P* Value |
| --- | --- | --- | --- |
| sFlt-1(pg/mL) | 5827.84 (4316.93, 9368.38) | 6021.58 (3457.59, 9763.89) | 0.764 |
| *PlGF(pg/mL) | 51.25 (37.97, 61.19) | 32.41 (26.60, 38.95) | <0.001 |
| ActivinA(pg/mL) | 865.30 (644.86, 1082.96) | 837.46 (553.93, 1090.85) | 0.750 |
| *sFlt-1/PlGF | 131.17 (81.74, 187.11) | 204.94 (103.33, 342.52) | 0.016 |

Note: Data are presented as the mean ± standard deviation or median (interquartile range). *P* < 0.05 was considered statistically significant and indicated by an asterisk.

Abbreviations: PlGF, placental growth factor; sFlt-1, soluble fms-like tyrosine kinase-1.

**Table S3. ROC analysis of the predictive/diagnostic value of early pregnancy biomarkers for PE**

| First-trimester Biomarkers | AUC (95% CI) | *P* value |
| --- | --- | --- |
| Total IgM | 0.878 (0.786-0.969) | <0.001 |
| Total IgG | 0.856 (0.767-0.946) | <0.001 |
| PlGF | 0.825 (0.726-0.924) | <0.001 |
| aPS IgG | 0.784 (0.675-0.893) | <0.001 |
| Total IgA | 0.769 (0.654-0.885) | <0.001 |
| RANTES | 0.737 (0.619-0.855) | 0.001 |
| sFlt-1/PlGF | 0.670 (0.539-0.800) | 0.011 |
| IL-2Rα | 0.660 (0.529-0.790) | 0.023 |
| HGF | 0.632 (0.497-0.767) | 0.039 |
| aPS-IgG IC | 0.632 (0.498-0.765) | 0.035 |
| IL-9 | 0.631 (0.499-0.763) | 0.037 |
| TNF-β | 0.630 (0.497-0.762) | 0.028 |

Abbreviations: AUC area under the curve; CI, confidence interval; PlGF, placental growth factor; aPS, anti-phosphatidylserine; Ig, immunoglobulin; IC, immune complex; sFlt-1, soluble fms-like tyrosine kinase-1; IL-2Rα, interleukin-2Rα; HGF, hepatocyte growth factor; IL-9, interleukin-9; TNF-β, tumor necrosis factor-β.

# Additional file Figure


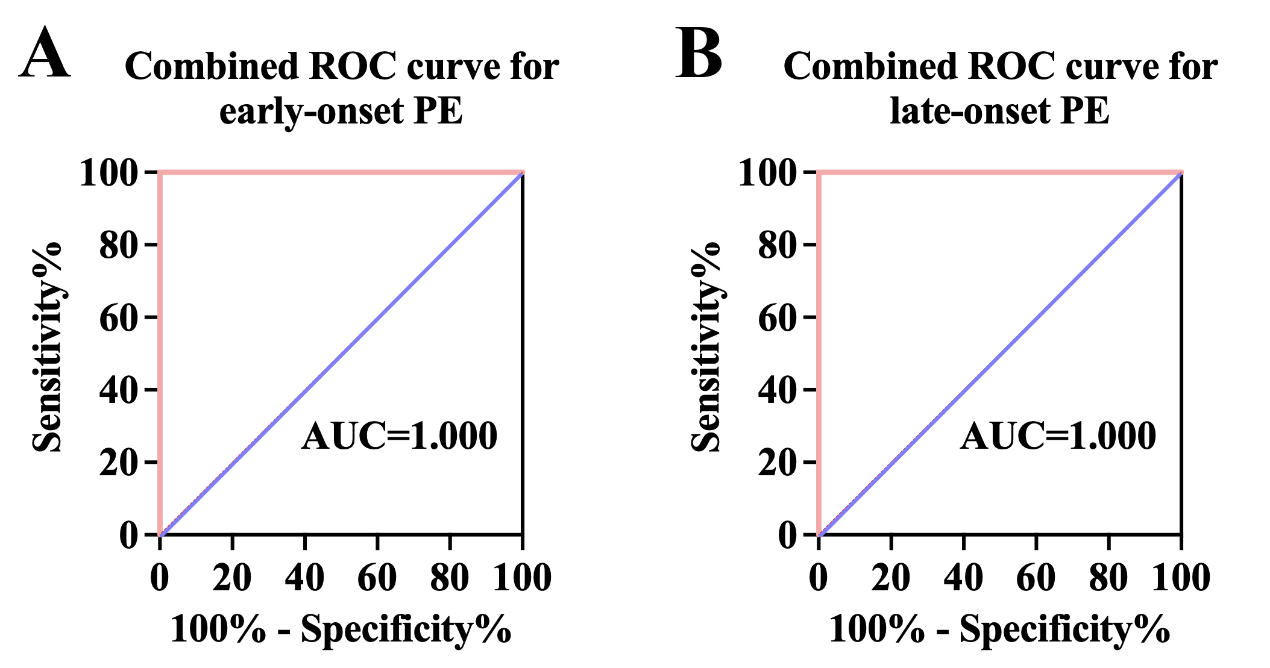


**Figure S1. The ROC curve for early- and late-onset PE.**

A, ROC curve for the combined analysis of total IgM, total IgG, PlGF, aPS IgG, and total IgA for predicting early-onset PE. B, ROC curve for the combined analysis of total IgM, total IgG, PlGF, aPS IgG, and total IgA for predicting late-onset PE.

ROC, receiver operating characteristic; AUC, area under the curve.
